# A Comprehensive Assessment Using Physicochemical and Microbial Indicators Reveals Enhanced Soil Health Under Integrated Rice-Red Swamp Crayfish (*Procambarus clarkii*) Farming

**DOI:** 10.3390/biology15070525

**Published:** 2026-03-25

**Authors:** Sihan Wang, Bing Li, Rui Jia, Linjun Zhou, Yiran Hou, Jian Zhu

**Affiliations:** 1 College of Fisheries and Life Science, Shanghai Ocean University, Shanghai 201306, China; lou1sewang@outlook.com; 2 Key Laboratory of Integrated Rice-Fish Farming Ecology, Ministry of Agriculture and Rural Affairs, Freshwater Fisheries Research Center, Chinese Academy of Fishery Sciences, Wuxi 214081, China; 3 Wuxi Fisheries College, Nanjing Agricultural University, Wuxi 214081, China

**Keywords:** integrated rice-red swamp crayfish farming, *Procambarus clarkii*, paddy field microorganisms, soil health index

## Abstract

Soil degradation poses a serious threat to the sustainability of global agricultural development, endangering the foundation and environment of human survival. Therefore, elucidating the effects of different agricultural production patterns on the quality and health of paddy soils is of great significance. To investigate the impact of the integrated rice-red swamp crayfish farming on paddy soil health, this paper systematically analyzed the differences in 19 soil physicochemical indicators and bacterial and eukaryotic microbial communities between the traditional rice monoculture (TRM) and integrated rice-red swamp crayfish (*Procambarus clarkii*) farming (IRPF), and it features a comprehensive quantitative assessment of paddy soil health status through Principal Component Analysis based on a minimum dataset. The experimental results showed that IRPF significantly increased the soil aggregate mean weight diameter, total phosphorus, available potassium, cation exchange capacity, pH, available zinc, and available silicon contents. Meanwhile, IRPF exerted marked effects on the beta diversity and composition of both bacterial and eukaryotic microbial communities, markedly enhancing the relative abundances of Bacillariophyta and Chlorophyta in the paddy soil. The integrated analysis of 19 soil physicochemical indicators along with bacterial and eukaryotic microbial community parameters revealed that the Soil Health Index under IRPF was obviously higher than that under the rice monoculture. In conclusion, the integrated rice-red swamp crayfish farming system markedly impacted the soil fertility, effectively improved soil aggregate structure and enhanced the overall paddy soil health status, representing a promising and sustainable agricultural production pattern within a single production cycle.

## Simple Summary

Soil degradation poses a severe threat to the sustainability of global agricultural development and endangers the foundation of human survival and the environment. This paper investigated the effects of an integrated rice-red swamp crayfish (*Procambarus clarkii*) farming system on the health status of paddy soil. We measured soil physical, chemical, and biological property indicators under both rice monoculture and the integrated rice-red swamp crayfish farming system, and we assessed the soil health status using the soil health index. The experimental results showed that the integrated rice-red swamp crayfish farming system significantly improved the soil aggregate structure and exerted a remarkable influence on soil fertility. Meanwhile, it notably increased the relative abundance of Bacillariophyta and Chlorophyta in paddy soil. The soil health index was significantly higher than that under the rice monoculture system, enhancing the overall health status of paddy soil. This paper demonstrates a promising sustainable agricultural production model within a single production cycle, providing a scientific basis for promoting the sustainable development of paddy ecosystems.

## 1. Introduction

Soil degradation is a severe challenge threatening global agricultural sustainability, manifesting as soil erosion, salinization, pollution, structural damage, and organic matter decline [1,2]. This process not only directly weakens soil productivity but also impairs key functions of soil ecosystems by altering the microbial community structure and diversity [3,4]. Therefore, soil health, as the core of maintaining ecosystem sustainability, environmental resilience, and agricultural productivity, has received increasing attention [5]. Different agricultural management practices have significant differences in their impacts on soil physical and chemical properties. Traditional intensive farming often leads to soil erosion and nutrient depletion, causing ecological environment degradation and making agricultural production systems unsustainable [6,7]. Conversely, conservation agriculture practices, such as reasonable cropping and husbandry systems and methods, have been proven to effectively improve soil structure, enhance fertility, and increase microbial diversity [8].

As an integrated farming model that combines rice cultivation with aquaculture, integrated rice-fish farming has demonstrated significant ecological and economic benefits [9]. Driven by the intrinsic material circulation process of the integrated rice-red swamp crayfish farming, the application amounts of chemical fertilizers and pesticides are significantly reduced. By doing so, the potential risk of agricultural diffuse pollution is lowered, and more favorable living conditions are created for the aquatic organisms in this specific ecosystem [10]. In 2024, the total area of the integrated rice-fish farming in China reached 307,020 hectares, accounting for 11% of the total rice planting area [11]. Among them, the integrated rice-red swamp crayfish (*Procambarus clarkii)* farming is the most widely used integrated rice-fish farming model in China. Existing studies have shown that the rice-crayfish co-culture model can effectively improve the soil aggregate structure, increase the soil nutrient content, and reshape the composition and functions of soil microbial communities. Long-term implementation of this model not only significantly alters the physicochemical properties of paddy soil and optimizes the bacterial community structure but also further enhances the species richness and diversity of bacteria in deep soil [12,13,14,15,16]. However, current studies lack quantitative assessments and in-depth discussions on the influences of integrated rice-red swamp crayfish farming on paddy soil health status.

Previous studies have summarized various soil health assessment systems for quantitatively evaluating soil health status [17,18]. Indicators related to physical, chemical and biological properties can reflect soil conditions and have been widely used in soil health-related studies [19,20,21,22,23,24]. Among them, physical indicators including bulk density (BD) and soil aggregate stability can reflect the soil structure and water retention capacity [25,26]; chemical indicators including the soil organic matter (SOM), total nitrogen (TN), total phosphorus (TP), available nitrogen (AN), available phosphorus (AP), available potassium (AK), cation exchange capacity (CEC), available selenium (Available Se), available zinc (Available Zn), and available silicon (Available Si) can reflect soil fertility and nutrient cycling and are important indicators for evaluating soil fertility [27,28,29,30,31,32,33,34,35]. Soil pH affects various biogeochemical and physical processes and is an important indicator of soil health and degradation [36]; biological indicators including microbial biomass carbon (MBC), microbial biomass nitrogen (MBN), amino sugars, microbial diversity, and relative abundance can reflect soil biological activity, material cycling, and ecological functions [37,38,39]. Based on this, this paper used traditional rice monoculture as a control to systematically investigate the influences of the integrated rice-red swamp crayfish farming on soil physicochemical characteristics and both bacterial and eukaryotic microbial communities in paddy fields. By constructing a soil health assessment index, this paper quantified soil quality differences under this mode, aiming to provide a scientific basis for optimizing integrated rice-red swamp crayfish farming management strategies and promoting the sustainable development of paddy field ecosystems.

## 2. Materials and Methods

### 2.1. Experiment Design and Sample Collection

This study was performed in 2024 at the Yangshan Research and Testing Base (120.08° E, 31.60° N, Wuxi, China) of the Freshwater Fisheries Research Center, Chinese Academy of Fishery Sciences. The experiment involved eight standardized rice fields, each with dimensions 8 m × 5 m and a total area of 40 m^2^. Four fields were used for traditional rice monoculture (TRM), while the other four were used for integrated rice-red swamp crayfish (*Procambarus clarkii)* farming (IRPF). The rice variety used was Nanjing 5055. Rice transplanting began on 22 July. On 26 July, 150 g of urea was applied, followed by 250 g of potassium sulfate compound fertilizer (containing total nitrogen ≥ 15%, available P_2_O_5_ ≥ 15%, and potassium chloride ≥ 15%) on 28 July, which was purchased from Fengmanlong Biotechnology Co., Ltd. (Changsha, China). An additional 500 g of urea was applied on August 3. On 16 August, red swamp crayfish were released into the four integrated rice-red swamp crayfish farming plots at a size of 6.245 ± 0.233 g per crayfish and a stocking density of 25 g·m^−2^, respectively. Feeding was conducted daily at 17:00 with commercial feed equivalent to 2% of the crayfish’s body weight. The *Procambarus clarkii* feed was purchased from Cargill Feed Co., Ltd. (Zhenjiang, China), which was composed of the following ingredients: crude protein > 32.0%, crude fat > 4.0%, crude ash < 20.0%, crude fiber < 11.0%, total phosphorus ≥ 0.8%, moisture ≤ 12.0%.

Harvesting and sampling were conducted on 30 October from 8:00 to 10:00. Surface soil samples were collected from five preselected points in each paddy field via the five-point sampling method and then thoroughly homogenized in a bucket. Each mixed soil sample, weighing approximately 200 g, was immediately placed in dry ice-filled containers for transport back to the laboratory, where they were preserved at a temperature of −80 °C. Physical, chemical, biological, and microbial analyses were conducted as soon as possible.

### 2.2. Soil Property Analysis and Measurement Methods

This experiment measured 2 physical indicators including BD and the soil aggregate mean weight diameter (MWD); 11 chemical indicators including SOM, TN, TP, AN, AP, AK, CEC, pH, available Se, available Zn, and available Si; as well as 6 biological indicators including MBC, MBN, glucosamine (GlcN), mannosamine (ManN), galactosamine (GalN), and muramic acid (Mur). The specific measurement methods are shown in Table S1 [40,41,42,43,44,45,46,47,48,49,50,51,52,53].

### 2.3. DNA Extraction and Metagenomic Sequencing

We extracted microbial DNA from soil samples by employing the E.Z.N.A.^®^ Soil DNA Kit (Omega Bio-tek, Norcross, GA, USA). The V3-V4 region of bacterial 16S rRNA was amplified using primers 341F (5′-CCTAYGGGRBGCASCAG-3′) and 806R (5’-GGACTACNNGGGTATCTAAT-3’), while eukaryotic microbial 18S rRNA was amplified using primers TAReuk454FWD1 (5’-CCAGCASCYGCGGTAATTCC-3’) and TAReukREV3 (5’-ACTTTCGTTCTTGATYRA-3’). The PCR products obtained after purification were subjected to NGS library construction, which was followed by high-throughput sequencing performed on the DNBSEQ-G99 platform at BGI (BGI Shenzhen Co., Ltd., Shenzhen, China) (processed by Shanghai Biozeron Biotechnology Co., Ltd., Shanghai, China).

Raw sequencing reads were subjected to quality control using FASTP version 0.20.0 [54] and merged using FLASH version 1.2 [55] with a minimum overlap length of 10 bp and a maximum mismatch rate of 2%. After removing duplicate sequences, the DADA2 algorithm in QIIME 2 (version 2020.11) was used to detect insertions-deletions and substitution mutations, defining amplicon sequence variants (ASVs). Paired-end sequencing reads were trimmed and filtered at a maximum expected error (maxEE) threshold of ≤2. The taxonomic classification of bacterial and eukaryotic microorganism ASVs was performed against the Silva and nt databases, respectively.

### 2.4. Statistical Analyses and Data Visualization

For the datasets of soil physicochemical factors, SPSS 27.0 was adopted to conduct an independent sample t-test for inter-group difference comparison with statistical significance determined at *p* < 0.05. In addition, graphs and figures were compiled using GraphPad Prism 9.0, Origin 2024 and Adobe Illustrator 2025 software.

The alpha diversity of soil microorganisms was assessed by calculating the Chao1 and Shannon diversity indices [56]. Principal Coordinates Analysis (PCoA) based on Bray-Curtis distance matrices was conducted to assess beta diversity [57]. The t-test was employed to compare discrepancies in the relative abundances of the top 10 dominant bacterial and eukaryotic microbial phyla between TRM and IRPF groups. It was also employed to assess statistically significant differences in microbial diversity indices among sample groups.

### 2.5. Soil Health Index (SHI) Calculation Method

#### 2.5.1. Determine Soil Health Evaluation Indicators and Construct a Minimum Data Set (MDS)

Principal Component Analysis (PCA) is a data dimensionality reduction technique that condenses multiple variables into a small number of core indicators via dimensionality reduction and serves as the basis for constructing the Minimum Dataset (MDS). First, principal components (PCs) with eigenvalues ≥ 1 are selected, and indicators with absolute loading values ≥ 0.5 on the same PC are grouped into one cluster. For indicators with absolute loading values ≥ 0.5 on multiple PCs, they are assigned to the group with the weakest correlation with other indicators. If an indicator has absolute loading values < 0.5 on all PCs, it is assigned to the group with the highest absolute loading value. Subsequently, the comprehensive loading (Norm value) of each indicator across all PCs is calculated according to Formula (1). Within each group, indicators with Norm values within the top 10% of the maximum value are selected, and the correlation between these selected indicators is analyzed. When the correlation coefficient is ≥0.5, only the indicator with the higher Norm value is included in the MDS; otherwise, if the correlation coefficient is <0.5, both indicators are incorporated into the MDS. In this paper, the Norm value reflects the vector length of each soil indicator in the multidimensional space formed by the principal components. A higher Norm value indicates greater cumulative loading of the variable across all PCs and stronger information interpretation ability. Using the Norm value-improved PCA method to screen indicators can avoid information loss issues [58,59].

(1) $$N_{ik}=\sqrt{\sum_{i=1}^{k}U_{ik}^{2}\lambda_{k}}$$

#### 2.5.2. Indicator Transformation, Weight Determination and Calculation of Soil Health Index (SHI)

We transformed the measured values of the indicators selected in the MDS into dimensionless numerical or categorical grades ranging from 0 to 1 by establishing membership functions, which represent the relative status of the corresponding attribute [60,61]. Two main types of standardized membership scoring functions were used: the positive S-shaped function represents the “the more, the better” criterion (Formula (2)), while the inverted S-shaped function represents the “the less, the better” criterion (Formula (3)) [62]. The coefficient of variation and weights of the membership function scores were calculated using Formulas (4) and (5). The Soil Health Index (SHI) was then computed by combining the membership function scores of the indicators with their corresponding weights (Formula (6)).

The coefficient of variation (also known as the variation coefficient), a statistic that reflects the degree of data dispersion, is used to directly and objectively assign weights to each indicator based on the information contained in the data [63]. A larger coefficient of variation corresponds to a greater weight assigned to the indicator. The SHI was calculated using weight coefficients, which aimed to address the unequal contributions of different soil evaluation indicators to soil health and assign meaningful weights to the selected indicators [64,65].

(2) $$f\left(x\right)=\left\{\begin{array}{c} \;\;\;\;\;\;\;\;\;\;0.1\;\;\;\;\;\;\;\;\;\;\;\;\;\;,X\leq X_{min}\\ 0.1+0.9\times \frac{X-X_{min}}{X_{max}-X_{min}},X_{min}<X<X_{max}\\ \;\;\;\;\;\;\;\;\;\;\;\;1.0\;\;\;\;\;\;\;\;\;\;\;\;\;\;,X\leq X_{max} \end{array}\right.$$

(3) $$f\left(x\right)=\left\{\begin{array}{c} 1\;\;\;\;\;\;\;\;\;\;\;\;\;\;\;\;\;\;\;\;\;\;\;\;\;\;\;,X\leq X_{min}\\ 1-0.9\times \frac{X-X_{min}}{X_{max}-X_{min}},X_{min}<X<X_{max}\\ 0.1\;\;\;\;\;\;\;\;\;\;\;\;\;\;\;\;\;\;\;\;\;\;\;\;\;,X\leq X_{max} \end{array}\right.$$

where X_min_ denotes the minimum value of the indicator, X_max_ represents the maximum value of the indicator, and X corresponds to any given value of the indicator [66].

(4) $$V_{i}=\frac{\sigma_{i}}{{\bar{x}}_{i}}$$

(5) $$W_{i=}\frac{V_{i}}{\sum V_{i}}$$

W_i_ represents the weight value of the i-th indicator, V_i_ represents the coefficient of variation in the i-th indicator, ΣV_i_ represents the sum of coefficients of variation in all indicators, σ_i_ signifies the standard deviation of the i-th indicator, while X_i_ represents the mean value of the i-th indicator.

(6) $$SHI=\sum_{i=1}^{n}W_{i}\times S_{i},$$

In this formula, SHI denotes the comprehensive evaluation index of soil health, i represents the number of evaluation indicators, W_i_ denotes the weight coefficient of the i-th indicator, and S_i_ corresponds to the membership score of the i-th evaluation indicator.

## 3. Results

### 3.1. Soil Properties

**Figure 1:**
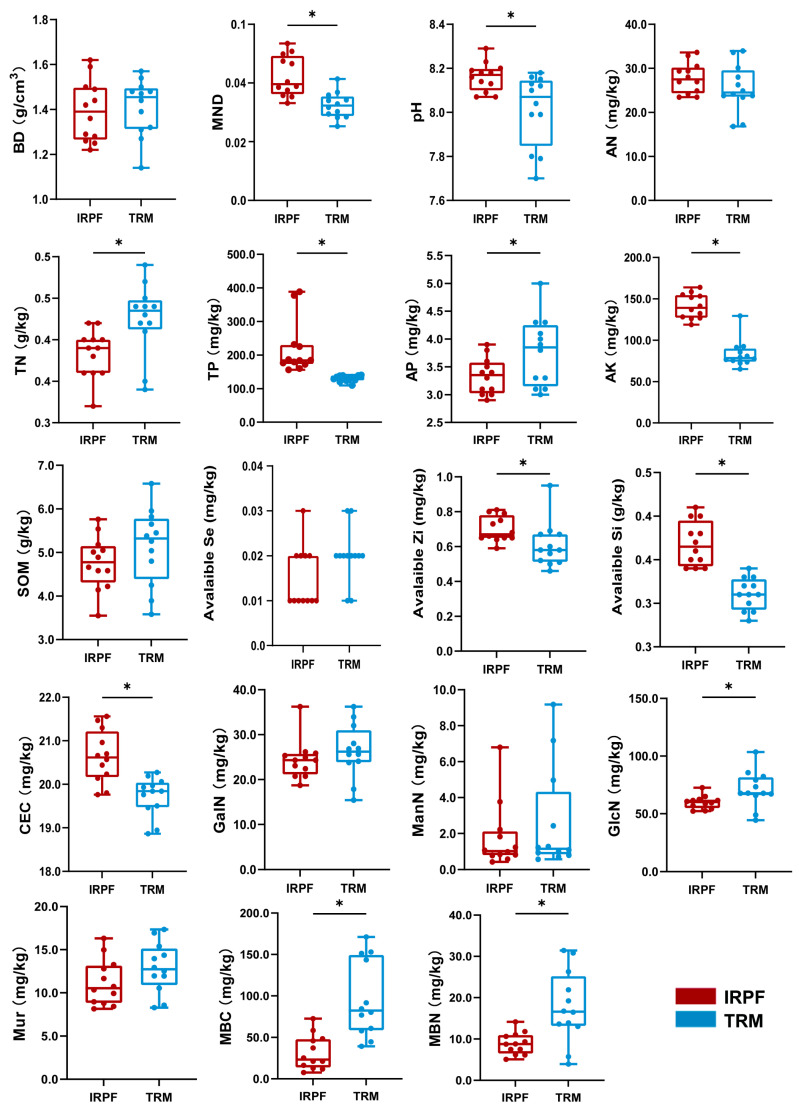
Soil physical, chemical, and biological characteristics including soil bulk density (BD, g∙cm^−3^), mean weight diameter (MWD), pH, available nitrogen (AN, mg∙kg^−1^), total phosphorus (TP, mg∙kg^−1^), available phosphorus (AP, mg∙kg^−1^), available potassium (AK, mg∙kg^−1^), total nitrogen (TN, g∙kg^−1^), soil organic matter (SOM, g∙kg^−1^), available selenium (Available Se, mg∙kg^−1^), available zinc (Available Zn, mg∙kg^−1^), available silicon (Available Si, g∙kg^−1^), cation exchange capacity (CEC, mg∙kg^−1^), glucosamine (GlcN, mg∙kg^−1^), galactosamine (GalN, mg∙kg^−1^), mannosamine (ManN, mg∙kg^−1^), muramic acid (Mur, mg∙kg^−1^), microbial biomass carbon (MBC, mg∙kg^−1^), and microbial biomass nitrogen (MBN, mg∙kg^−1^) within paddy soil between the IRPF and TRM groups. Significant differences (*p* < 0.05) between IRPF and TRM are indicated by an asterisk (*).

As illustrated in Figure 1, the soil BD of the IRPF group was 1.393 ± 0.040 g∙cm^−3^, while that of the TRM group was 1.409 ± 0.037 g∙cm^−3^, showing no significant difference (*p* > 0.05). The MWD of the IRPF group was 0.042 ± 0.002 compared to 0.032 ± 0.001 for the TRM group.

As opposed to the TRM group, the IRPF group demonstrated significant increments in soil chemical indicators, including TP, AK, CEC, pH, Available Zn, and Available Si, while significant decreases were observed in TN and AP (*p* < 0.05). There were no significant differences in SOM, AN, and Available Se (*p* > 0.05). The soil biological indicators were GlcN, ManN, GalN, Mur acid, MBC, and MBN. Of these parameters, the IRPF group exhibited significantly lower GlcN, MBC and MBN levels than the TRM group (*p* < 0.05), whereas no statistically notable variations were detected in ManN, GalN and Mur between the two groups (*p* > 0.05).

### 3.2. Soil Microbial Communities

**Figure 2:**
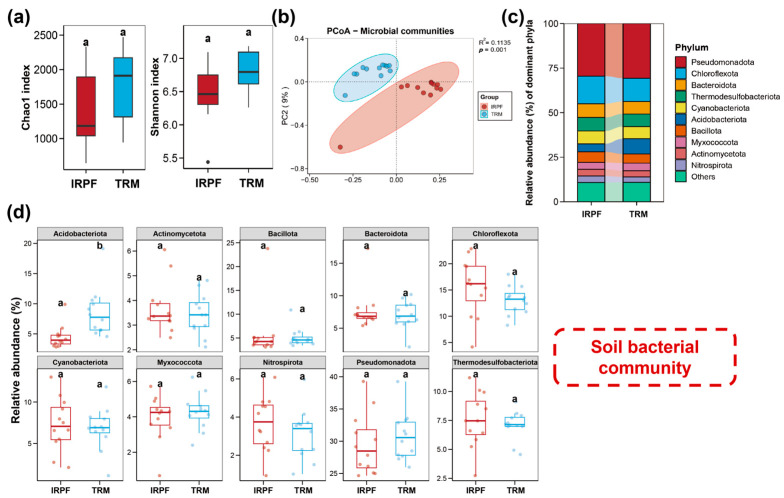
Bacterial communities in soil across the IRPF and TRM groups. (**a**) The Chao1 index and Shannon index of bacteria in soil from the IRPF group and the TRM group. (**b**) Beta diversity of bacteria in soil from the IRPF group and TRM group. (**c**) The major bacterial phyla (the top 10 with the highest abundance) in soil across IRPF and TRM groups. (**d**) Top 10 variations in phylum-level bacterial species composition and relative abundance between IRPF and TRM groups. Different letters indicate significant differences among different groups (*p* < 0.05).

**Figure 3:**
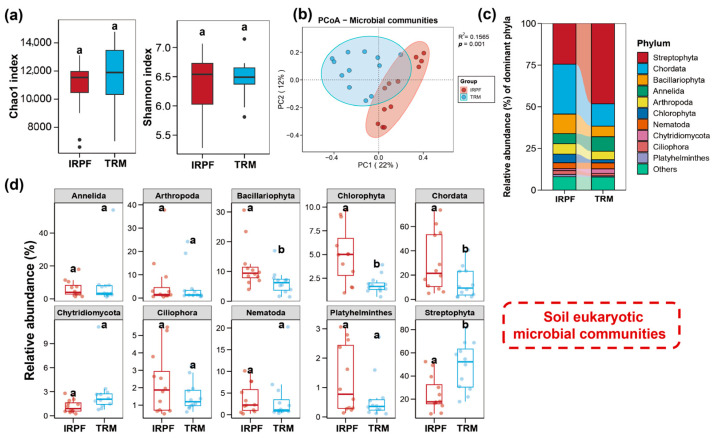
Eukaryotic microbial communities in soil of the IRPF group and TRM group. (**a**) Chao1 index and Shannon index of eukaryotic microorganisms in soil of the IRPF group and TRM group. (**b**) Beta diversity of eukaryotic microbial communities in soils of IRPF group and TRM group. (**c**) The predominant eukaryotic microbial phyla (the top 10 with the highest abundance) in soil of the IRPF group and TRM group. (**d**) Top 10 differences in eukaryotic microbial species composition and relative abundance at the phylum level between the IRPF group and TRM group. Statistically significant intergroup differences (*p* < 0.05) are denoted by distinct lowercase letters above the respective bars.

The phylum-level composition of soil bacterial communities is presented in Figure 2c. The major phyla of bacterial in both IRPF and TRM groups were similar, which were mainly composed of Pseudomonadota, Chloroflexota, Bacteroidota, Thermodesulfobacteriota, Cyanobacteriota, and Acidobacteriota, accounting for more than 70% of the total bacteria. Among them, Pseudomonadota exhibited the maximum relative abundance in both the IRPF and TRM groups, while the relative abundance of Acidobacteriota in the IRPF group was notably lower than that in the TRM group (*p* < 0.05; Figure 2d). The compositional characteristics of the top 10 eukaryotic microbial phyla are shown in Figure 3c; these phyla were dominated by Streptophyta, Chordata, Bacillariophyta, Annelida, Arthropoda, and Chlorophyta, constituting over 70% of the total eukaryotic microbial. Among them, compared with the TRM group, the IRPF group presented significantly higher relative abundances of Bacillariophyta, Chlorophyta, and Chordata (*p* < 0.05), whereas the relative abundance of Streptophyta in the IRPF group registered a markedly greater decrease (*p* < 0.05).

As presented in Figure 2a,b and Figure 3a,b, no notable variations (*p* > 0.05) were detected in the alpha diversity (Shannon index and Chao1 index) of soil bacterial and eukaryotic microbial communities between the IRPF and TRM groups. However, significant variations were detected in the beta diversity of both the soil bacterial and eukaryotic microbial communities in the two groups of soil samples (*p* < 0.05).

**Figure 4:**
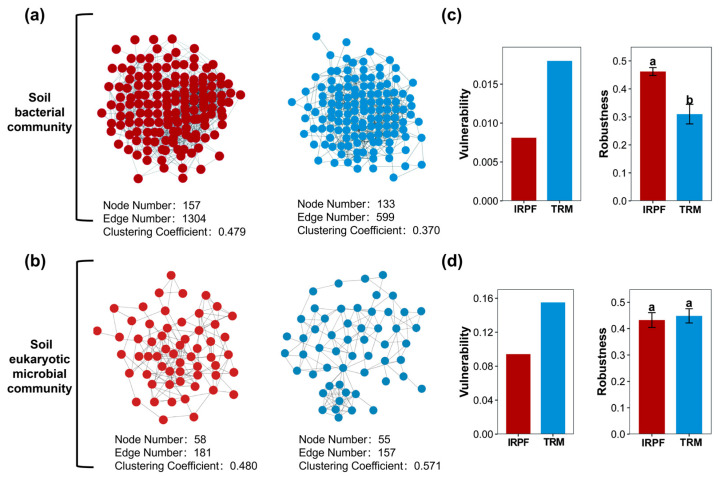
The co-occurrence networks for the bacterial and eukaryotic microbial community in soil across IRPF and TRM groups. Differences in co-occurrence networks of bacterial (**a**) and eukaryotic microbial communities (**b**) across both IRPF and TRM groups, including nodes, edges, and clustering coefficient. Differences in robustness and vulnerability of bacterial (**c**) and eukaryotic microbial communities (**d**) across the IRPF and TRM groups. Different letters indicate significant differences among different groups (*p* < 0.05).

As shown in Figure 4a, the co-occurrence networks of soil bacterial and eukaryotic microbial communities in the IRPF group were more complex (in terms of nodes and edges) than those in the TRM group. In the bacterial network, the IRPF group contained 157 nodes and 1304 edges, while the TRM group contained 133 nodes and 599 edges. In the eukaryotic microbial network, the IRPF group contained 58 nodes and 181 edges, while the TRM group contained 55 nodes and 157 edges. The network clustering coefficients for bacteria and eukaryotic microbes were 0.479 and 0.480 in the IRPF group as opposed to 0.370 and 0.571 in the TRM group, respectively.

Additionally, the robustness index of soil bacterial communities in the IRPF group exhibited a notably greater elevation than that in the TRM group (Figure 4c, *p* < 0.05), and the vulnerability index was inferior to that in the TRM group. The vulnerability index of the soil eukaryotic microbial communities in the IRPF group was also depressed relative to that in the TRM group. This indicates that the soil bacterial community network stability in the IRPF group was stronger than that in the TRM group.

### 3.3. Soil Health Index Calculation Results

In summary, a total of 19 soil physicochemical indicators were included in the analysis. Meanwhile, referring to the microbial indicators selected by Zhao (2024) [67], we further included taxa with significantly different relative abundances in bacterial and eukaryotic microbial communities in this paper, including Acidobacteriota, Bacillariophyta, Chlorophyta, and Streptophyta, as well as the Shannon index (an indicator of microbial diversity) and Chao1 index (a measure of microbial richness) derived from alpha-diversity analysis. A total of 27 indicators were used for subsequent analysis.

**Figure 5:**
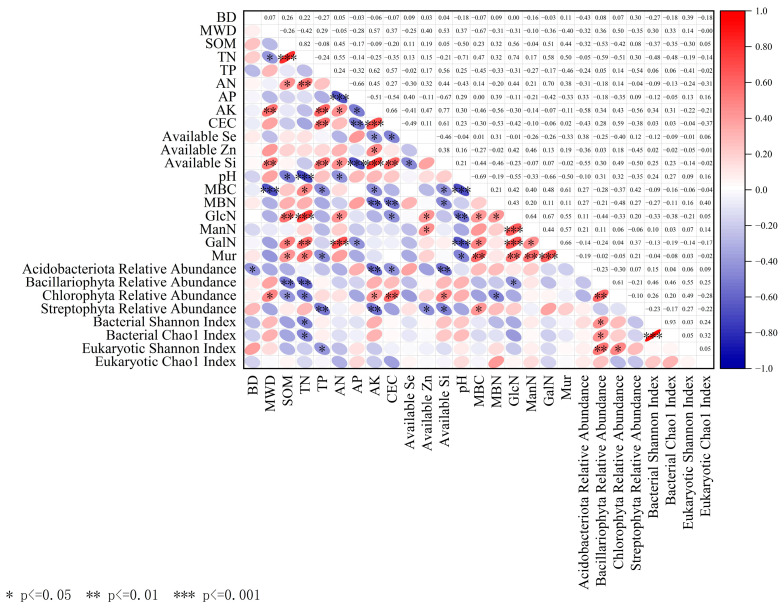
Correlation coefficient matrix plot of the 27 indicators. The upper right section displays the corresponding correlation coefficients. In the lower left section, negative correlations are indicated using blue circles, and positive correlations are marked with red circles. Darker colors indicate stronger correlations with asterisks (*) denoting significance (*p* < 0.05).

We performed PCA on 27 indicators (Table 1). Based on the criterion of eigenvalue > 1, seven PCs were retained. First, the indicators were grouped according to the absolute values of factor loadings and correlation requirements. Then, Norm values were calculated using Formula (1), and the top 10% of indicators with the highest values in each group were retained. At this stage, the candidate indicators were as follows: Principal Component 1 (PC1) included TN, GlcN, and Available Si; Principal Component 2 (PC2) included AK; Principal Component 3 (PC3) included the relative abundance of Bacillariophyta, TP, and the bacterial Shannon index; Principal Component 4 (PC4) included the relative abundance of Streptophyta; Principal Component 5 (PC5) included Available Zn; Principal Component 6 (PC6) included the bacterial Chao1 index. No indicators meeting the criteria were found in Principal Component 7 (PC7). Subsequently, correlation analysis and comparison were conducted on the selected indicators in each principal component (Figure 5). Therefore, the final indicators incorporated into the MDS were TN, GlcN, AK, relative abundance of Bacillariophyta, TP, bacterial Shannon index, relative abundance of Streptophyta, Available Zn, and bacterial Chao1 index. Finally, the selected indicators were standardized and normalized using membership functions (Formulas (2) and (3)), and the weights of each evaluation indicator were determined by the coefficient of variation method (Formulas (4) and (5)) with the results shown in Table 2. The average SHI of the IRPF group was 0.511, which was significantly higher than that of the TRM group (0.404) (*p* < 0.05), and the relevant data are presented in Table 3.

## 4. Discussion

### 4.1. Effects of Integrated Rice-Red Swamp Crayfish Farming on Soil Properties

Soil serves as the substrate for plant growth, and its physicochemical properties are vital environmental indicators. In this paper, the MWD of soil aggregates in the IRPF group exhibited a notable elevation compared with that in the TRM group, while the BD was lower. Soil aggregates constitute the fundamental units of soil structure. Generally, higher values of Geometric Mean Diameter (GMD) and MWD indicate greater aggregate stability [68]. This observation can be ascribed to the enhancement of the soil aggregate structure induced by the integrated rice-crayfish farming, since the burrowing behaviors of soil macrofauna are capable of decreasing soil BD and elevating soil porosity [69]. The results indicate that compared to the TRM group, the IRPF group exhibited significant increases in soil pH, AK, TP, Available Zn, Available Si, and CEC. TN and TP represent the soil’s nutrient supply potential, whereas available nutrients such as AK and AP reflect the soil’s immediate nutrient supply capacity [70]. Similar to previous studies, in the integrated rice-fish farming systems, the input of exogenous feed and the bioturbation caused by aquaculture animals are likely the primary factors influencing environmental variables in paddy fields [71,72,73]. On one hand, as typical benthic organisms, red swamp crayfish can significantly promote the degradation of organic matter in the soil, thereby enhancing the elemental cycling processes between the soil and water [74,75]. On the other hand, the input of feed and the accumulation and decomposition of uneaten feed and feces in integrated rice-fish farming systems substantially affect the nutrient element contents in the paddy soil and water [73]. Based on this, we hypothesized that the changes in nutrient element contents within paddy soil in this study resulted from the combined effects of red swamp crayfish bioturbation and exogenous feed input with this integrated effect significantly influencing soil fertility. In addition, field management practices can influence the contents of MBC and MBN in integrated rice-fish farming system [76,77]. Soil pH serves as a pivotal factor in regulating soil fertility. Previous research has demonstrated that the pH level in the integrated rice-crayfish farming system is notably higher than that in the rice monoculture system, which is a result that aligns with the conclusions drawn from this paper [78]. Furthermore, pH tends to increase cumulatively with the duration of aquaculture [79]. The significant increase in soil Available Zn within the IRPF group effectively promoted plant growth and development [80].

Overall, the integrated rice-red swamp crayfish farming system improved soil aggregate structure and exerted a significant influence on soil fertility.

### 4.2. Effects of Integrated Rice-Red Swamp Crayfish Farming on Soil Microorganisms

Microorganisms residing in soil perform a central function in sustaining soil nutrient cycling processes and structural stability. Their community composition and abundance are jointly modulated by soil factors including pH, temperature, and nutrient content [81,82,83,84]. With respect to the compositional characteristics of soil microbial communities, although the IRPF group showed no notable variations in the indices of alpha diversity compared to the TRM group, the microbial community structure exhibited significant divergence. In terms of bacterial composition at the phylum level, the relative abundance of Acidobacteriota in the IRPF group exhibited a marked reduction relative to that in the TRM group. Acidobacteriota can secrete diverse extracellular acidic substances, regulate biogeochemical cycles, and influence plant growth [85,86]. At the phylum level of eukaryotic microorganisms, the IRPF group exhibited a markedly higher relative abundance of Bacillariophyta and Chlorophyta in comparison with the TRM group. Bacillariophyta and Chlorophyta play crucial roles in phosphorus assimilation, nitrogen fixation, soil amelioration, and the promotion of rice growth [87]. Therefore, the significant changes in the dominant phyla of soil bacterial and eukaryotic microbial communities in the integrated rice-red swamp crayfish farming might indicate their impact on soil nutrient cycling and transformation processes mediated by Acidobacteriota, Bacillariophyta, and Chlorophyta, which in turn affects soil health and rice growth.

Through the quantification of nodes and edges, microbial co-occurrence networks are capable of revealing intricate inter-microbial interactions and the stability of microbial communities [88,89,90]. Furthermore, the stability of the microbial co-occurrence network can be assessed using robustness and vulnerability metrics [91,92]. In this paper, the co-occurrence network of the soil bacterial community in the IRPF group possessed more nodes and edges than that of the TRM group, indicating a greater number of interactions. Concurrently, the robustness of the bacterial community in the IRPF group was significantly enhanced, while its vulnerability significantly decreased. No statistically significant variations were identified in the eukaryotic microbial community. Collectively, these results indicate that the integrated rice-red swamp crayfish farming gives rise to a soil bacterial community with higher complexity and persistence [93,94].

### 4.3. Effects of Integrated Rice-Red Swamp Crayfish Farming on Soil Health Status

Since 2020, the significance of soil biodiversity has garnered increasing attention within the academic and research communities. However, owing to the limited understanding of functional attributes and the absence of effective methodological approaches, the quantification of soil health has remained predominantly reliant on chemical indicators [95]. Microorganisms play a crucial ecological role in soil, and their diversity, along with the relative abundance of dominant taxa, is essential for soil functioning. Yet, these aspects are often overlooked in traditional soil health evaluations [96,97]. This paper incorporated microbial parameters into the assessment of paddy soil health status. Our findings indicated that adopting the integrated rice-red swamp crayfish farming system positively influenced paddy field health within a single production cycle. Yuan et al. (2020) [98] also confirmed that the rice-red swamp crayfish enhanced the paddy soil health by an assessment of physicochemical and biological indicators. This improvement may be attributed to the burrowing, crawling, and foraging activities of crayfish, which effectively improve the soil’s physical permeability. Additionally, the continuous return of residual feed and crayfish excrement to the field serves as high-quality organic fertilizers [99,100]. This finding is consistent with our results.

Nevertheless, some studies have suggested that the long-term monoculture of the same species may lead to land degradation and that the SHI varies depending on crop rotation practices [60,101,102]. However, this paper did not conduct long-term field experiments; soil samples were collected only once at the rice harvest stage. Therefore, it cannot reveal the long-term cumulative effects of the integrated rice-red swamp crayfish farming on soil health. Consequently, in future research, increasing sampling replication and extending the experimental duration are crucial and necessary to further evaluate the long-term effects of the integrated rice-red swamp crayfish farming on the health status of paddy soil ecosystems.

## 5. Conclusions

In conclusion, compared with rice monoculture, the integrated rice-red swamp crayfish farming system significantly altered the physical, chemical, and biological properties of paddy soil, exerted a significant impact on soil fertility, and improved soil aggregate structure. Simultaneously, this model significantly affected the beta diversity and the compositional profiles of bacterial and eukaryotic microbial communities alike, markedly increasing the relative abundances of Bacillariophyta and Chlorophyta. Furthermore, by integrating indicators related to soil characteristics with those associated with bacterial and eukaryotic microbial communities, a comprehensive assessment of the SHI was conducted. The results showed that the integrated rice-red swamp crayfish farming increased the Soil Health Index within a single production cycle; however, its long-term sustained effects warrant further investigation. Therefore, increasing sampling replication and extending the experimental duration to assess the long-term impacts of this integrated farming model on the health status of paddy soil ecosystems will be a key focus of future research.

## Figures and Tables

**Table 1:** Principal Component Analysis for selecting minimum dataset based on 27 soil indicators. (underlined to indicate selection).

Indicators	PC 1	PC2	PC3	PC4	PC5	PC6	PC7	Norm
TN	−0.776	0.463	−0.218	−0.009	0.125	0.123	0.122	2.391
GlcN	−0.717	0.440	0.108	0.269	0.278	−0.229	0.154	2.285
Available Si	0.654	0.604	−0.069	0.068	0.081	0.170	−0.064	2.256
CEC	0.644	0.456	−0.093	−0.210	−0.269	−0.195	−0.253	2.121
pH	0.678	−0.405	−0.120	0.064	0.220	−0.177	−0.117	2.101
MBC	−0.707	0.111	0.341	0.038	−0.401	0.044	−0.160	2.099
Chlorophyta Relative Abundance	0.643	0.242	0.387	−0.403	−0.013	−0.225	0.130	2.058
SOM	−0.554	0.490	−0.345	0.059	0.282	0.360	−0.071	2.044
MWD	0.715	0.113	0.004	−0.021	0.352	−0.009	0.318	2.040
MBN	−0.578	−0.282	0.064	0.157	0.450	0.063	−0.107	1.829
AK	0.737	0.542	−0.142	0.118	0.013	0.091	0.078	2.366
GalN	−0.506	0.681	0.253	0.002	−0.162	0.035	0.068	2.121
AN	−0.111	0.865	−0.068	0.034	−0.125	0.076	0.207	2.008
Mur	−0.469	0.549	0.489	−0.018	0.003	0.052	−0.267	1.980
AP	−0.235	−0.763	−0.127	0.041	0.223	−0.153	−0.031	1.892
Acidobacteriota Relative Abundance	−0.323	−0.548	0.170	0.335	−0.340	−0.288	0.278	1.757
Available Se	−0.303	−0.511	−0.343	0.058	0.063	0.168	0.365	1.595
Bacillariophyta Relative Abundance	0.603	−0.080	0.582	−0.160	0.152	0.102	−0.174	1.953
TP	0.558	0.248	−0.512	0.285	−0.160	−0.013	−0.059	1.895
Bacterial Shannon Index	0.476	−0.112	0.523	0.416	−0.183	0.386	0.275	1.810
ManN	−0.281	0.321	0.631	0.345	0.149	−0.423	0.030	1.682
Eukaryotic Shannon Index	0.090	-0.266	0.536	-0.567	0.316	-0.071	0.065	1.490
Streptophyta Relative Abundance	−0.547	−0.141	0.202	−0.535	−0.181	0.175	0.163	1.798
Eukaryotic Chao1 Index	−0.015	−0.295	0.229	0.512	0.390	0.319	−0.420	1.362
BD	−0.114	0.099	−0.036	−0.667	0.443	0.320	0.095	1.326
Available Zn	0.175	0.489	0.026	0.297	0.625	−0.334	0.198	1.625
Bacterial Chao1 Index	0.473	−0.170	0.475	0.375	−0.077	0.498	0.211	1.784
Eigenvalue	7.324	5.138	2.848	2.393	1.979	1.443	1.027	
Variance explained (%)	27.125	19.029	10.547	8.863	7.330	5.345	3.804	
Cumulative variance explained (%)	27.125	46.154	56.701	65.564	72.894	78.240	82.044	

**Table 2:** Standardized values and weights of membership functions for selected indicators.

Group	TN	GlcN	AK	Bacillariophyta Relative Abundance	TP	Bacterial Shannon Index	Streptophyta Relative Abundance	Available Zn	Bacterial Chao1 Index
IRPF	0.524	0.413	0.589	0.180	0.339	0.944	0.220	0.449	0.840
	0.471	0.360	0.662	0.341	0.493	0.643	0.229	0.724	0.349
	0.312	0.331	0.655	0.444	0.348	1.000	0.299	0.449	1.000
	0.418	0.348	0.904	0.274	1.000	0.568	0.106	0.339	0.448
	0.312	0.219	0.951	0.786	0.249	0.984	0.484	0.431	0.890
	0.312	0.317	0.794	0.357	0.302	0.985	0.639	0.467	0.987
	0.629	0.360	0.675	0.379	0.473	0.524	0.219	0.449	0.248
	0.524	0.223	0.890	0.398	0.262	0.770	0.373	0.706	0.711
	0.524	0.292	0.918	0.318	0.965	0.829	0.184	0.633	0.737
	0.629	0.373	1.000	0.251	0.342	0.759	0.100	0.743	0.683
	0.100	0.248	0.713	1.000	0.315	0.860	0.207	0.504	0.752
	0.471	0.529	0.754	0.310	0.326	0.728	0.602	0.596	0.377
TRM	1.000	0.729	0.686	0.154	0.179	0.559	0.226	0.522	0.273
	0.788	0.631	0.349	0.100	0.180	0.747	1.000	0.357	0.690
	0.894	0.677	0.340	0.331	0.161	0.888	0.461	0.486	0.792
	0.735	0.426	0.243	0.106	0.200	0.472	0.665	0.210	0.272
	0.629	0.455	0.193	0.275	0.133	0.580	0.838	0.173	0.303
	0.735	0.454	0.286	0.279	0.192	0.511	0.838	0.320	0.315
	0.259	0.100	0.176	0.301	0.151	0.721	0.358	0.100	0.556
	0.206	0.169	0.100	0.273	0.196	0.865	0.384	0.192	0.791
	0.576	0.445	0.212	0.226	0.196	0.651	0.713	0.284	0.387
	0.682	1.000	0.205	0.178	0.100	0.605	0.276	1.000	0.346
	0.629	0.457	0.230	0.586	0.153	0.953	0.606	0.320	0.931
	0.735	0.541	0.166	0.219	0.147	0.100	0.748	0.486	0.100
Mean	0.546	0.421	0.529	0.336	0.308	0.718	0.449	0.456	0.574
Standard Deviation	0.222	0.197	0.303	0.205	0.232	0.211	0.259	0.208	0.271
Coefficient of Variation	0.407	0.469	0.574	0.609	0.751	0.294	0.576	0.457	0.473
SUM COV	4.611								
Weight	0.088	0.102	0.124	0.132	0.163	0.064	0.125	0.099	0.102

**Table 3:** The Soil Health Index of IRPF and TRM groups.

Group	SHI	Mean
IRPF	0.459	0.511
	0.463	
	0.506	
	0.513	
	0.570	
	0.545	
	0.434	
	0.514	
	0.604	
	0.511	
	0.514	
	0.503	
TRM	0.441	0.404
	0.498	
	0.504	
	0.347	
	0.374	
	0.416	
	0.277	
	0.319	
	0.383	
	0.435	
	0.497	
	0.352	

## Data Availability

The raw data supporting the conclusions of this paper are available from the corresponding author upon reasonable request.

## Supplementary Materials

The following supporting information can be downloaded at https://www.mdpi.com/article/10.3390/biology15070525/s1, Table S1: Soil property analysis and measurement methods.

## Abbreviations

The following abbreviations are used in this manuscript:

IRPF	Integrated rice-red swamp crayfish *Procambarus clarkii* farming
TRM	Traditional rice monoculture
AK	Available potassium
TN	Total nitrogen
TP	Total phosphorus
AN	Available nitrogen
AP	Available phosphorus
SOM	Soil organic matter
CEC	Cation exchange capacity
MBC	Microbial biomass carbon
MBN	Microbial biomass nitrogen
Mur	Muramic acid
GlcN	Glucosamine
GalN	Galactosamine
ManN	Mannosamine
MWD	Mean weight diameter
BD	Bulk density
MDS	Minimum Dataset
SHI	Soil Health Index
PCA	Principal Component Analysis
