# Supplementary material for: A Comprehensive Assessment Using Physicochemical and Microbial Indicators Reveals Enhanced Soil Health Under Integrated Rice-Red Swamp Crayfish (Procambarus clarkii) Farming
Source: Biology (Basel). 2026 Mar 25;15(7):525. doi: 10.3390/biology15070525 (PMC13072144; doi:10.3390/biology15070525)
Supplement: Supplementary file 1 [file biology-15-00525-s001.zip › Table S1.pdf]

Table S1. Soil property analysis and measurement methods.

Classification	Category	Measurement Method
Physics	Bulk density (BD)	Soil Testing Part 4: Method for determination of soil bulk density [16]
	Mean weight diameter (MWD)	Soil Testing Part 3: Method for determination of soil mechanical composition [17]
Chemistry	Soil organic matter (SOM)	Method for determination of soil organic matter [18]
	Total nitrogen (TN)<br>Available nitrogen （AN）	Nitrogen determination methods of forest soils [19]
	Total phosphorus (TP)	Soil-Determination of Total Phosphorus by alkali fusion-Mo-Sb Anti spectrophotometric method [20]
	Available phosphorus (AP)	Soil quality-Determination of available phosphorus-Sodium hydrogen carbonate solution-Mo-Sb anti spectrophotometric method [21]
	Available potassium (AK)	Determination of exchangeable potassium and non-exchangeable potassium content in soil [22]
	Cation exchange capacity (CEC)	Soil quality-Determination of cation exchange capacity (CEC) Hexamminecobalt trichloride solution-Spectrophotometric meth [23]
	pH	Determination of pH in Soil [24]
	Available selenium (Available-Se)	Determination of available selenium in soil-Hydride generation atomic fluorescence spectrometry [25]
	Available zinc (Available-Zn)	Determination of available zinc, manganese, iron, copper in soil-extraction with buffered DTPA solution [26]
	Available silicon (Available-Si)	Soil Testing Part 15: Method for determination of soil available silicon [27]
Biology	Microbial biomass carbon (MBC)<br>Microbial biomass nitrogen (MBN)	Determination of soil microbial biomass-Fumigation-extraction method [28]
	Aminosugars	Gas chromatography-hydrogen flame ionization detection (GC-FID) [29]
